# Force-field-based minimizations of protein-ligand complexes in the blink of an eye

**DOI:** 10.1186/1758-2946-5-S1-P14

**Published:** 2013-03-22

**Authors:** Lennart Heinzerling, Matthias Rarey

**Affiliations:** 1 Center for Bioinformatics, University of Hamburg, Hamburg, Germany

## 

Basing protein-ligand optimizations on molecular mechanics force fields yields high accuracy at the cost of speed. We address this dilemma by circumventing unnecessary calculations and by harvesting the computational power of graphics processor units (GPUs). This novel computational architecture is well suited for parallelizing and massively speeding up force-field-based minimization procedures. Our endeavor encompasses fundamental adaptions of formerly CPU-based methods in order to take the GPU-specific programming paradigms into account. This results in force-field kernels uniquely targeted at the efficient optimization of protein-ligand complexes in the context of a mostly GPU-based procedure. This procedure constitutes an improvement of our recently published method[1] which focuses on complex optimizations with rigid proteins. The new version presented here features side chain flexibility in the protein's active site. Additionally, it allows to guide the optimization procedure by considering pharmacophoric-type geometric constraints. In this way, we exploit knowledge about binding modes and conformational preferences. In detail, preferred dihedral angles are annotated to rotatable bonds and selected key hydrogen bond interactions are marked and conserved.

We demonstrate the procedure's potential by performing a large-scale docking run on the Astex-non-native set[2]. By optimizing the resulting protein-ligand complexes, we significantly shift the more promising conformations towards the crystal structure geometry of the complex. In spite of using the full point-to-point potential, an optimization run for a protein-ligand complex finishes in less than 500 ms on average.
